# Recovery of response and long-term outcomes following loss of response and dose escalation of subcutaneous infliximab: a post hoc analysis of the LIBERTY-CD & LIBERTY-UC trials

**DOI:** 10.1093/ibd/izag017

**Published:** 2026-03-18

**Authors:** Marla C Dubinsky, Stefan Schreiber, Andres J Yarur, Bruce E Sands, Stephen B Hanauer, Silvio Danese, Hyunseong Yu, Dong-Hyeon Kim, Young Nam Lee, Jean-Frédéric Colombel

**Affiliations:** Department of Pediatrics, Susan and Leonard Feinstein Inflammatory Bowel Disease Clinical Center, Icahn School of Medicine at Mount Sinai, New York, NY, United States; Department of Medicine I, University Hospital Schleswig-Holstein, Kiel University, Kiel, Germany; F. Widjaja Inflammatory Bowel Disease Institute, Cedars Sinai Medical Center, Los Angeles, CA, United States; Dr Henry D. Janowitz Division of Gastroenterology, Icahn School of Medicine at Mount Sinai, New York, NY, United States; Division of Gastroenterology and Hepatology, Department of Medicine, Northwestern University, Feinberg School of Medicine, Chicago, IL, United States; Department of Gastroenterology, IRCCS San Raffaele Hospital and Vita-Salute University, Milan, Italy; Global Medical Division, Celltrion Inc., Incheon, Republic of Korea; Global Medical Division, Celltrion Inc., Incheon, Republic of Korea; Global Medical Division, Celltrion Inc., Incheon, Republic of Korea; Dr Henry D. Janowitz Division of Gastroenterology, Icahn School of Medicine at Mount Sinai, New York, NY, United States

**Keywords:** subcutaneous infliximab, dose escalation, Crohn’s disease, ulcerative colitis, loss of response

## Abstract

**Background:**

Rapidity of onset of efficacy following dose escalation of subcutaneous infliximab after loss of response remains unclear in Crohn’s disease (CD) and ulcerative colitis (UC). This post hoc analysis of the LIBERTY-CD and LIBERTY-UC trials evaluated time to response recovery following dose escalation of subcutaneous infliximab after loss of response, and characterized patients who experienced early recovery.

**Methods:**

This analysis included week 10 responders to intravenous infliximab induction who were randomized to receive subcutaneous infliximab 120 mg every other week (Q2W) and underwent dose escalation to 240 mg Q2W following loss of response. Time to response recovery was assessed, and outcomes pre-/post-dose escalation were analyzed by recovery timing (early [≤8 weeks], late [>8 weeks], non-recovery). Week 102 outcomes and factors associated with early recovery were evaluated.

**Results:**

Response recovery was achieved in 85.1% (40/47) with CD and 82.3% (51/62) with UC, with early recovery in 66.0% (31/47) and 69.4% (43/62), respectively. Early recovery groups in CD and UC showed greater serum infliximab increases than late or non-recovery groups. At week 102, numerically higher rates of clinical response and clinical remission in CD, and endoscopic remission in UC, were observed in early versus late recovery group. Factors associated with early recovery differed between CD and UC: systemic inflammatory and pharmacokinetic parameters were linked to early recovery in CD, while mucosal and gut-specific factors predominated in UC.

**Conclusion:**

Dose escalation of subcutaneous infliximab led to rapid response recovery in most patients with CD and UC. Early recovery was associated with favorable long-term outcomes.

**Clinical trial registration numbers:**

NCT03945019 and NCT04205643.

## Graphical abstract

**Graphical Abstract:**
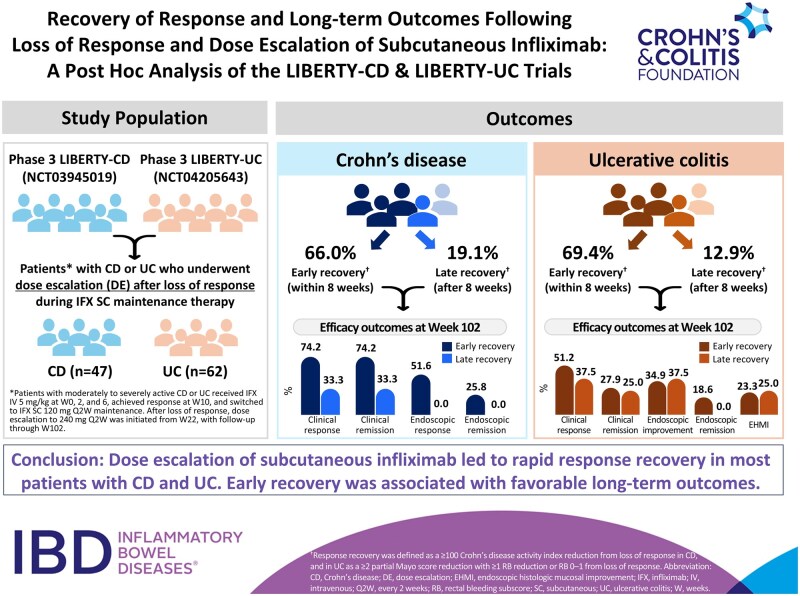

Key Messages **What is already known?** 

- Dose escalation of intravenous infliximab is an established approach to rapidly restore response, whereas initial evidence supporting the benefits of dose escalation of subcutaneous infliximab is emerging.

**What is new here?** 

- Dose escalation of subcutaneous infliximab led to regaining response within 8 weeks in the majority of patients, and these patients showed favorable long-term outcomes; however, factors associated with the rapidity of regaining response vary between Crohn’s disease and ulcerative colitis.

**How can this study help patient care?** 

- The feasibility of treatment optimization is reaffirmed for patients who experienced loss of response during maintenance therapy with subcutaneous infliximab, offering insights into the rapidity and likelihood of regaining response following dose escalation.

## Introduction

Infliximab (IFX) is a monoclonal antibody that binds with high affinity to soluble and transmembrane forms of tumor necrosis factor alpha (TNF-α), and is an established treatment for moderately to severely active Crohn’s disease (CD) and ulcerative colitis (UC).^1–3^ Subcutaneous IFX (IFX SC), a new formulation of IFX, was approved as a new drug by the U.S. Food and Drug Administration (FDA) in 2023 for the maintenance treatment of moderately to severely active CD and UC following intravenous (IV) induction after demonstrating superiority over placebo in the phase 3 LIBERTY-CD and -UC trials.^4^^,^^5^ IFX SC enables dosing at intervals approximating the antibody’s half-life, unlike IV bolus administration, which leads to significantly more stable serum concentrations.^6^^,^^7^

Some patients with CD and UC experience a loss of response to IFX IV treatment over time, with an estimated discontinuation rate of 20-40% during the maintenance phase.^8^^,^^9^ In these patients, dose escalation (DE) is a common strategy for managing secondary loss of response by improving serum IFX concentrations and restoring clinical efficacy.^9–13^ By optimizing drug exposure, DE is important for reducing underlying inflammation, thereby improving clinical symptoms and decreasing the need for hospitalization and surgery.^9–12^

IFX SC was also approved by the European Medicines Agency as a maintenance treatment at a dose of 120 mg every other week (Q2W) for patients with CD and UC following the demonstration of pharmacologic and clinical equivalence to IFX IV.^14^^,^^15^ While the FDA approved IFX SC only at a dose of 120 mg Q2W, in Europe, an intensified dose of 240 mg Q2W was additionally approved for patients with CD who initially responded to induction therapy with IFX but subsequently lost response.^15^

Given the utility of DE in managing loss of response to IFX IV,^16–18^ interest has grown in exploring the potential benefits of DE in ­patients receiving IFX SC, with reference to treat-to-target recommendations.^19^ Initial analyses of the phase 3 LIBERTY studies demonstrated that DE with IFX SC effectively restores clinical efficacy and provides sustained long-term benefit without additional safety concerns.^20^ Although a few real-world studies have reported clinical improvements after DE of IFX SC, these data are limited to small patient numbers.^21–24^ Additionally, to date, no study has specifically detailed the rapidity of response following IFX SC DE, its association with long-term outcomes, or identified predictive factors associated with early recovery in patients who met predefined loss of response criteria. Analyzing and understanding these ­aspects may offer critical insights into the clinical utility and optimal use of IFX SC in patients with inflammatory bowel disease (IBD).

The present post hoc analysis of the LIBERTY-UC and LIBERTY-CD studies uniquely explores the rapidity of response recovery after IFX SC DE in patients who experienced loss of response, its association with long-term treatment targets, and clinical or pharmacokinetic predictors of early recovery.

## Methods 

### Study design and treatment

This study is a post hoc analysis of data from LIBERTY-CD and LIBERTY-UC trials (LIBERTY-CD: NCT03945019; LIBERTY-UC: NCT04205643). Detailed eligibility criteria for the original trials have been previously reported.^4^^,^^5^ Briefly, the trials enrolled patients aged 18-75 years with moderately to severely active CD or UC, who had an inadequate response to corticosteroids and immunomodulators. Patients received induction therapy with IFX IV (5 mg/kg) at weeks 0, 2, and 6. Clinical responders at week 10 (defined as a reduction of 100 points or more from the baseline Crohn’s Disease Activity Index [CDAI] score for patients with CD; a 2-point and 30% reduction from baseline in the modified Mayo score [MMS], with a 1-point reduction in the rectal bleeding subscore [RB] or an absolute RB of 0-1 for patients with UC) were randomized (2:1) to receive either IFX SC 120 mg or placebo Q2W as maintenance therapy.

### Analysis population

DE with IFX SC (240 mg Q2W) was initiated from week 22 in patients who initially responded but subsequently lost response. Loss of response was defined as a CDAI increase of ≥100 points from week 10, resulting in a total CDAI of ≥220 for patients with CD; an increase in the MMS of ≥2 points and ≥30% from week 10, with an actual value of ≥5 points and a Mayo Endoscopic Subscore (MES) of ≥2 points for patients with UC. The present post hoc study only included patients with CD and UC who met predefined criteria for loss of response in the randomized IFX SC treatment arm and subsequently underwent DE.

### Study outcomes

Response recovery after DE was defined in patients with CD as a reduction of ≥100 points in CDAI from the time of loss of response. In patients with UC, response recovery was defined as a decrease in partial Mayo score (PMS) from loss of response of at least 2 points, with an accompanying decrease in the RB of at least 1 point, or an absolute RB of 0 or 1.

CDAI, PMS, Short Inflammatory Bowel Disease Questionnaire (SIBDQ) outcomes, and pharmacokinetic levels were compared at pre- and post-DE. Pre-DE was defined as the week the patient experienced loss of response for CDAI/PMS and SIBDQ outcomes, or as the week of DE for pharmacokinetic evaluation. Post-DE was defined as the first visit after DE, which was used as the time point for assessing CDAI/PMS following DE and could be a scheduled visit, an unscheduled visit, or the end of study visit.

Patient outcomes were also analyzed based on time to response recovery. The early recovery group was defined as patients who achieved response recovery within 8 weeks after DE, while the late recovery group was defined as patients who achieved response recovery after 8 weeks. The non-recovery group was defined as patients who were censored before response recovery. Subgroup analyses for pharmacokinetics and efficacy were performed by measuring CDAI and SIBDQ scores in patients with CD, PMS and SIBDQ scores in patients with UC, and serum IFX concentrations in all patients.

The proportion of patients achieving efficacy outcomes at week 102 of the study was evaluated. In the CD study, clinical response was defined as a decrease in CDAI of ≥100 points from baseline, while clinical remission was defined as an absolute CDAI of <150 points. Endoscopic response was determined as a ≥50% reduction in the Simplified Endoscopic Activity Score for CD (SES-CD) from baseline, whereas endoscopic remission was defined as an absolute SES-CD of ≤4, with a reduction of ≥2 points from baseline and no subscore >1. In the UC study, clinical response was ­defined as a decrease of ≥2 points and ≥30% in the MMS from baseline, with an accompanying decrease of ≥1 point in the RB or an absolute RB of 0 or 1. Clinical remission was defined as an MMS where stool frequency and MES were 0 or 1, and the RB was 0. Endoscopic-histologic mucosal improvement was defined as an absolute MES of 0 or 1 in the MMS and a Robarts Histopathology Index score of ≤3, with both lamina propria neutrophils and epithelial neutrophils subscores of 0. Endoscopic improvement was defined as a MES of ≤1, while endoscopic remission was defined as an MES of 0. Corticosteroid-free remission was evaluated in patients who were receiving oral corticosteroids at baseline with the definition of clinical remission without receiving any corticosteroids for ≥8 weeks prior to week 102 in both CD and UC.

### Statistical analysis

Variables following a normal distribution, as determined by the Shapiro-Wilk test, are presented as mean (± standard deviation [SD]), while non-normally distributed variables are presented as median (interquartile range [IQR]). Time to DE was calculated by determining the difference between baseline and the week when patients underwent DE. Comparisons between pre-DE and post-DE outcomes were calculated using the paired Wilcoxon signed-rank test. For comparisons of delta values between groups, the Wilcoxon rank-sum test was used. Descriptive statistical analyses were generated using the mytable function (method 3) in the moonBook package in R (version 4.3.2). A nominal *P*-value < .05 was considered statistically significant.

To evaluate potential predictors of early recovery, univariable logistic regression analyses were performed for each variable. For missing data, last observation carried forward imputation was applied prior to performing logistic regression. Variables with a nominal *P*-value < .3 in the univariable analyses were selected for inclusion in an adjusted multivariable logistic regression model. The adjusted model was optimized using stepwise selection, implemented through the step function in R, applying both forward and backward selection to identify the most parsimonious model. Logistic regression analyses were performed using the generalized linear model (glm) function (family = binomial) in R (version 4.3.2) and values were presented as an adjusted odds ratio (OR; 95% confidence interval [CI]). As approximately half of the patients with CD and the majority of patients with UC experienced loss of response at week 22, a univariable analysis for week 22 was not performed.

## Results

### Characteristics of patients who underwent DE following loss of response

In the LIBERTY-CD and UC trials, 20.3% (47/231) and 21.1% (62/294) of patients in the SC arm underwent DE following loss of response and were included in the present analysis (Figure S1—see online supplementary material). Baseline patient demographics and clinical characteristics of these patients are presented in Table 1. The median (IQR) age at baseline was 35.0 (29.0-40.5) years in patients with CD and 36.0 (30.0-49.0) years in patients with UC. At baseline, patients with CD had a mean (SD) disease duration of 4.4 (4.2) years with a mean CDAI (SD) score of 325.5 (59.3) and SES-CD of 12.5 (6.4). Patients with UC had a mean (SD) disease duration of 7.9 (6.3) years, a mean (SD) MMS of 6.7 (1.1) and MES of 2.7 (0.5), with 19 (30.6%) and 43 (69.4%) patients having an MES of 2 and 3, respectively. At baseline, 5 (10.6%) patients with CD and 14 (22.6%) patients with UC had prior use of biologics or Janus kinase inhibitors (JAKi). Twenty-four (51.1%) patients with CD and 31 (50.0%) patients with UC had used oral corticosteroids at baseline. The median (IQR) time to DE following secondary loss of response was 38 (22-54) weeks in LIBERTY-CD and 22 (22-42) weeks in LIBERTY-UC (Figure S2—see online supplementary material). The median (IQR) follow-up period after DE was 45 (16-69) and 52 (30-84) weeks, respectively.

**Table 1:** Baseline characteristics of patients undergoing dose escalation following loss of response.

Baseline characteristics		CD (*n* = 47)	UC (*n* = 62)
**Age, median (IQR)**	Years	35.0 (29.0-40.5)	36.0 (30.0-49.0)
**Sex, *n* (%)**	Male	27 (57.4)	33 (53.2)
**Race, *n* (%)**	White	39 (83.0)	61 (98.4)
**Baseline BMI, mean (SD)**	kg/m^2^	24.3 (4.8)	23.8 (4.0)
**Disease duration, mean (SD)**	Years	4.4 (4.2)	7.9 (6.3)
**Disease location (CD)/extent (UC), *n* (%)**	Colonic/E1	27 (57.4)	9 (14.5)
	Ileocolonic/E2	15 (31.9)	28 (45.2)
	Ileal/E3	5 (10.6)	25 (40.3)
**Prior biologics or JAKi, *n* (%)**	Used	5 (10.6)	14 (22.6)
**Oral corticosteroids at baseline, *n* (%)**	Used	24 (51.1)	31 (50.0)
**Immunosuppressant (AZA, 6-MP, MTX) at baseline, *n* (%)**	Used	20 (42.6)	15 (24.2)
**Albumin at baseline, mean (SD)**	g/L	43.5 (4.0)	43.9 (4.0)
**Baseline CDAI, mean (SD)**	–	325.5 (59.3)	–
**MMS score, mean (SD)**	–	–	6.7 (1.1)
**Baseline SES-CD (CD), mean (SD)**	–	12.5 (6.4)	–
**MES (UC), mean (SD)**	–	–	2.7 (0.5)
**CRP, mean (SD)**	nmol/L	159.2 (247.0)	108.4 (149.4)
**FC, mean (SD)**	mg/kg	4,488.5 (11,484.0)	3,167.9 (3,791.3)
**SIBDQ score at baseline, mean (SD)**	–	34.8 (9.7)	33.6 (10.9)

Abbreviations: AZA, azathioprine; BMI, body mass index; CDAI, Crohn’s Disease Activity Index; CD, Crohn’s disease; CRP, C-reactive protein; FC, fecal calprotectin; IQR, interquartile range; JAKi, Janus kinase inhibitor; MES, Mayo endoscopic score; MMS, modified Mayo score; 6-MP, 6-mercaptopurine; MTX, methotrexate; SD, standard deviation; SES-CD, Simplified Endoscopic Score for Crohn’s Disease; SIBDQ, Short Inflammatory Bowel Disease Questionnaire; UC, ulcerative colitis.

### Clinical profile pre- and post-DE

**Figure 1:**
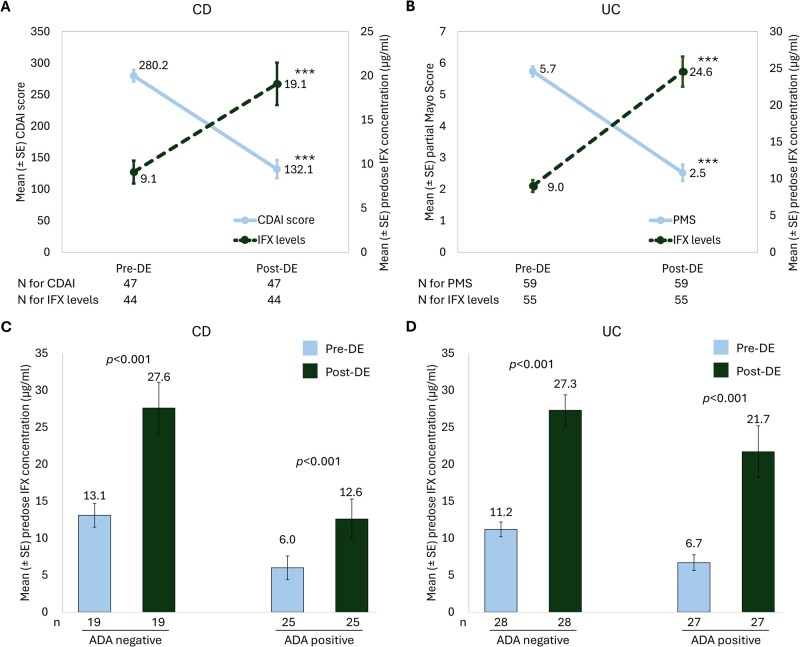
Impact of dose escalation on disease activity and serum drug levels. (A) Patients with Crohn’s disease, (B) Patients with ulcerative colitis, (C) Serum infliximab levels pre- and post-dose escalation by antidrug antibody status in patients with Crohn’s disease, (D) Serum infliximab levels pre- and post-dose escalation by antidrug antibody status in patients with ulcerative colitis. Pre-DE refers to the time of loss of response, which is the week when patients experience a loss of response. Post-DE refers to the first visit after DE, representing the time points for assessing CDAI/PMS following DE, including not only scheduled visits but also unscheduled visits and end of study visit. ***P < .001. Analyses were conducted on paired samples descriptively, and the nominal *P*-values were calculated by comparing Pre-DE and Post-DE data using the paired Wilcoxon signed-rank test. Abbreviations: ADA, antidrug antibodies; CDAI, Crohn’s Disease Activity Index; CD, Crohn’s disease; DE, dose escalation; IFX, infliximab; PMS, partial Mayo score; SE, standard error; UC, ulcerative colitis.

Following DE, patients experienced an overall improvement in disease activity with a reduction in the mean CDAI score in patients with CD from 280.2 to 132.1 (*P* < .001) and PMS in patients with UC from 5.7 to 2.5 (*P* < .001; Figure 1A and B). In addition, the mean serum IFX concentration increased from 9.1 µg/mL to 19.1 µg/mL in patients with CD (*P* < .001), and from 9.0 µg/mL to 24.6 µg/mL in patients with UC (*P* < .001; Figure 1A and B). When analyzed by antidrug antibody status, DE led to a significant increase of serum IFX levels in both antidrug antibody negative patients with CD (13.1 µg/mL to 27.6 µg/mL [*P* < .001]) and antidrug antibody positive patients (6.0 µg/mL to 12.6 µg/mL [*P* < .001]), although the magnitude of increase was greater in antidrug antibody negative patients (*P* = .009; Figure 1C). In patients with UC, both antidrug antibody negative (11.2 µg/mL to 27.3 µg/mL [*P* < .001]) and positive (6.7 µg/mL to 21.7 µg/mL [*P* < .001]) patients experienced a significant increase in serum IFX levels after DE (Figure 1D), with similar magnitudes of increase between groups (*P* = .311). No new safety concerns related to dose escalation were identified (Table S1—see online supplementary material).

### Characteristics of patients by time to response recovery

**Figure 2:**
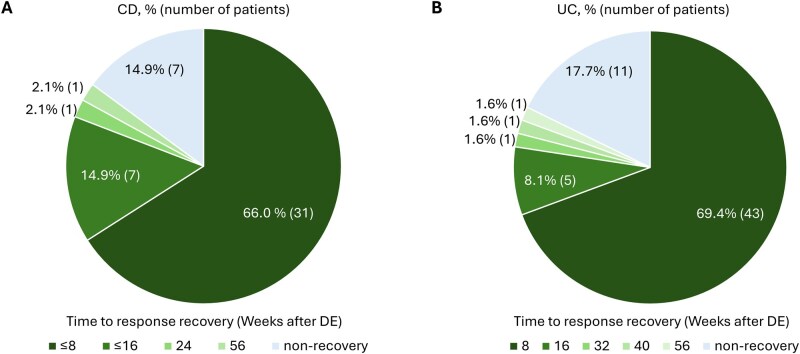
Time to response recovery after dose escalation in patients with Crohn’s disease (A) and ulcerative colitis (B). Patients were tested for their CDAI score or PMS on an 8-weekly basis. However, the timing of the relative visit may vary for some patients due to unscheduled DE visits. To conduct the time-to-event type analysis in patients with UC, the analysis was conducted using the PMS with a short efficacy measurement interval (8-weeks term). Response recovery for CD: CDAI-100 from loss of response; Response recovery for UC: Decrease in PMS from loss of response of at least 2 points, with an accompanying decrease in the RB of at least 1 point, or an absolute RB of 0 or 1 point. Abbreviations: CDAI, Crohn’s Disease Activity Index; CD, Crohn’s disease; DE, dose escalation; PMS, partial Mayo score; RB, rectal bleeding subscore; UC, ulcerative colitis.

Response recovery after DE was observed in 85.1% (40/47) of patients with CD and 82.3% (51/62) of patients with UC. 66.0% (31/47) of patients with CD and 69.4% (43/62) of patients with UC achieved response recovery within 8 weeks (early recovery group; Figure 2A and B and Figure S3—see online supplementary ­material). Additional 7, 1, and 1 patients with CD achieved recovery by weeks 16, 24, and 56, respectively, and 5, 1, 1, and 1 patients with UC by weeks 16, 32, 40, and 56, respectively (late recovery group). Seven and 11 patients with CD and UC, respectively, did not recover their response (non-recovery group).

Patient demographics and clinical characteristics were generally comparable between early, late and non-recovery groups for both CD and UC (Tables S2 and S3—see online supplementary material). In patients with CD, the rate of antidrug antibody positive conversion before loss of response was significantly different between groups, with the early recovery group showing a lower conversion rate relative to late or non-recovery groups (48.4%, 88.9%, and 85.7%, respectively; *P* = .033). In patients with UC, MMS at week 22 (5.0, 5.5, and 7.0, respectively; *P* = .035), MMS at the time of loss of response (6.0, 5.5, and 7.0, respectively; *P* = .005) and C-reactive protein (CRP) at baseline (31.4 nmol/L, 56.6 nmol/L, and 7.6 nmol/L, respectively; *P* = .031) showed significant differences between early, late and non-recovery groups. Additional patient characteristics by time of response recovery are listed in Table S4 (see online supplementary material).

### Pharmacokinetics by time to response recovery

**Figure 3:**
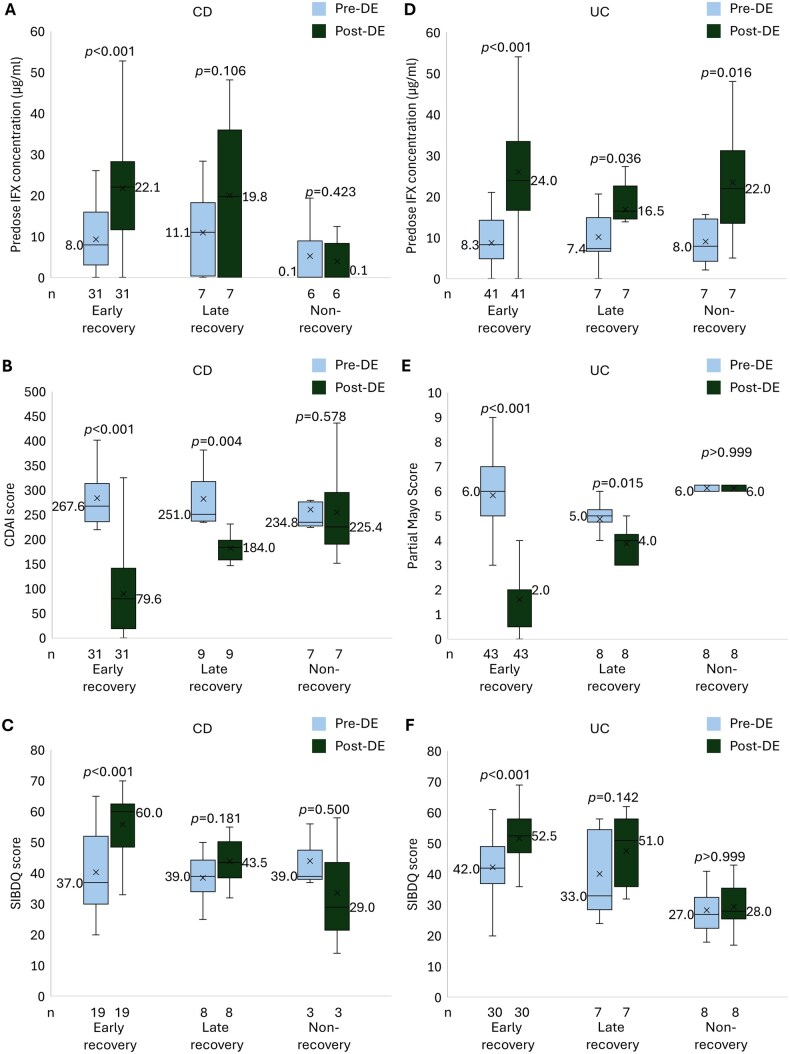
Impact of dose escalation on serum infliximab levels, disease activity, and quality of life by response recovery status. (A) Crohn’s disease—predose IFX concentration, (B) Crohn’s disease—CDAI score, (C) Crohn’s disease—SIBDQ score, (D) Ulcerative colitis—predose IFX concentration, (E) Ulcerative colitis—partial Mayo score, (F) Ulcerative colitis—SIBDQ score. The box plots display the median (line within the box), mean (×), interquartile range (IQR; box from Q1 to Q3), and the minimum and maximum values excluding outliers (whiskers). Pre-DE refers to the time of loss of response, defined as the week when patients experienced a loss of response for assessing CDAI (Crohn’s disease), PMS (ulcerative colitis), and SIBDQ, or the week of DE for assessing infliximab (IFX) concentration. Post-DE refers to the first visit after DE, representing the time points for assessing CDAI or PMS following DE, including not only scheduled visits but also unscheduled visits and end of study visit. Analyses were conducted on paired samples descriptively, and the nominal *P*-values were calculated by comparing Pre-DE and Post-DE data using the paired Wilcoxon signed-rank test. Abbreviations: CDAI, Crohn’s Disease Activity Index; CD, Crohn’s disease; DE, dose escalation; IFX, infliximab; IQR, interquartile range; PMS, partial Mayo score; SIBDQ, Short Inflammatory Bowel Disease Questionnaire; UC, ulcerative colitis.

In subgroup analysis by time to response recovery, median serum IFX concentration in patients with CD significantly increased in the early recovery group from 8.0 µg/mL to 22.1 µg/mL (*P* < .001), with a median increase of 10.9 µg/mL (Figure 3A). Although serum IFX concentration increased in the late recovery group (11.1 µg/mL to 19.8 µg/mL, median increase of 8.7 µg/mL, *P* = .106), these increases were not significant, while the non-recovery group showed no change in serum IFX concentration pre- and post-DE (0.1 µg/mL vs 0.1 µg/mL, *P* = .423; Figure 3A). In patients with UC, median serum IFX concentration significantly increased in the early recovery group (8.3 µg/mL to 24.0 µg/mL, *P* < .001, median increase of 14.6 µg/mL), late recovery group (7.4 µg/mL to 16.5 µg/mL, *P* = .036, median increase of 7.6 µg/mL), and the non-recovery group (8.0 µg/mL to 22.0 µg/mL, *P* = .016, median increase of 9.9 µg/mL; Figure 3D).

### Disease activity and quality of life by time to response recovery

In patients with CD, reductions in median CDAI score following DE were larger in the early recovery group (267.6 to 79.6 [*P* < .001]) relative to the late recovery group (251.0 to 184.0 [*P* = .004]; *P* < .007) and the non-recovery group (234.8 to 225.4 [*P* = .578]; *P* < .001), but not between the late recovery and non-recovery groups (*P* = .213; Figure 3B). In addition, median SIBDQ scores for the early recovery­ group increased significantly (37.0 to 60.0 [*P* < .001]), but not in the late recovery group (39.0 to 43.5 [*P* = .181]) and non-recovery group (39.0 to 29.0 [*P* = .500]; Figure 3C). Similarly, in patients with UC, reductions in median PMS following DE were larger in the ­early recovery group (6.0 to 2.0 [*P* < .001]) relative to the late recovery group (5.0 to 4.0 [*P* = .015]) and the non-recovery group (6.0 to 6.0 [*P* > .999]; *P* < .001 for both), and the late recovery relative to the non-recovery group (*P* = .015; Figure 3E). In addition, SIBDQ scores for the early recovery group (42.0 to 52.5 [*P* < .001]) were significantly increased following DE, but not in the late recovery group (33.0 to 51.0 [*P* = .142]) and non-recovery group (27.0 to 28.0 [*P* > .999]; Figure 3F). Changes in stool frequency and abdominal pain (CD) or RB (UC) from pre- to post-DE were significantly different between groups (Table S5—see online supplementary material). In addition, immunogenicity status, albumin levels, and CRP levels according to time to response recovery are presented in Tables S6 and S7 and Figure S4 (see online supplementary material).

### Efficacy outcomes at week 102 by time to response recovery

**Figure 4:**
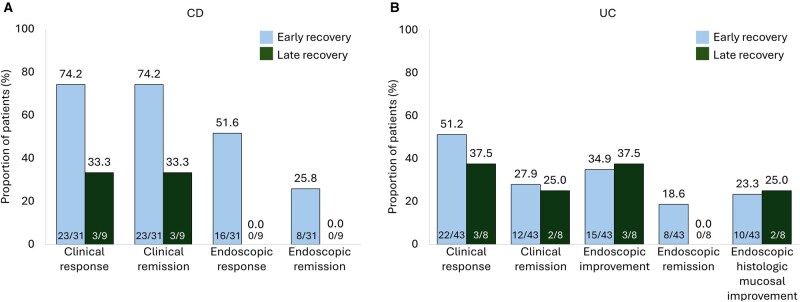
Clinical endpoints achieved at week 102 in patients with Crohn’s disease (A) or ulcerative colitis (B). In the CD study, clinical response: a reduction of 100 points or more from the baseline CDAI; clinical remission: a CDAI of <150; endoscopic response: a ≥50% reduction in the SES-CD from baseline; endoscopic remission: an absolute SES-CD of ≤4, with a ≥2-point reduction from baseline, and no subscore >1. In the UC study, clinical response: a ≥2-point and ≥30% reduction from baseline in the MMS, along with a ≥1-point reduction in the RB or absolute RB of 0-1; clinical remission: an MMS where stool frequency and MES were 0 or 1, with a RB of 0; endoscopic improvement: MES of ≤1; endoscopic remission (normalization): MES of 0; endoscopic-histologic mucosal improvement: an absolute MES of 0 or 1 in the MMS, alongside a Robarts Histopathology Index score of ≤3, with both lamina propria neutrophils and epithelial neutrophil subscores of 0. Abbreviations: CDAI, Crohn’s Disease Activity Index; CD, Crohn’s disease; MES, Mayo endoscopic score; MMS, modified Mayo score; RB, rectal bleeding subscore; SES-CD, Simplified Endoscopic Score for Crohn’s Disease; UC, ulcerative colitis.

At week 102, 74.2% of the early recovery group with CD achieved both clinical response and clinical remission, compared to 33.3% of the late recovery group (Figure 4A). Endoscopic response and endoscopic remission were observed in 51.6% and 25.8% of the early recovery group, respectively. None of the late recovery group achieved endoscopic response or endoscopic remission (Figure 4A). In patients with UC, 51.2% of the early recovery group achieved clinical response, compared to 37.5% of the late recovery group (Figure 4B). Clinical remission was observed in 27.9% of the early recovery group and 25.0% of the late recovery group. Although endoscopic improvement (34.9% vs 37.5%) and endoscopic-histologic mucosal improvement (23.3% vs 25.0%) were similar between the early and late recovery groups, none of the late recovery group achieved endoscopic ­remission, whereas 18.6% of the early recovery group did (Figure 4B). Corticosteroid-free remission was evaluated only in patients with baseline corticosteroid use. In patients with CD, remission was achieved in 11/16 (68.8%) of the early recovery group and 2/4 (50.0%) of the late recovery group. In patients with UC, remission was achieved in 6/23 (26.1%) of the early recovery group and 1/2 (50.0%) of the late recovery group.

### Multiple logistic regression analysis for early recovery

Logistic regression analyses were performed to further examine the association between patient characteristics and the likelihood of recovering response early (Table 2). In patients with CD, the adjusted analysis revealed that a positive antidrug antibody conversion prior to loss of response (adjusted OR [95% CI]: 0.04 [0.00-0.53], *P* = .014) and prior use of biologics or JAKi (adjusted OR [95% CI]: 0.03 [0.00-0.94], *P* = .046) were significantly associated with a lower likelihood of early recovery. Additionally, higher CRP levels at week 10 (adjusted OR [95% CI]: 3.20 [1.29-7.96], *P* = .012) and higher serum IFX concentration at week 10 (adjusted OR [95% CI]: 1.25 [1.04-1.50], *P* = .015) were positively associated with early recovery. In patients with UC, a longer time to DE (adjusted OR [95% CI]: 3.01 [1.12-8.06], *P* = .028) and higher fecal calprotectin levels at baseline (adjusted OR [95% CI]: 3.40 [1.03-11.30], *P* = .045) were significantly associated with a higher likelihood of early recovery. Conversely, the greater difference in MMS between week 10 and the week of loss of response (adjusted OR [95% CI]: 0.48 [0.24-0.95], *P* = .036) was associated with a lower likelihood of early recovery.

**Table 2:** Multivariable logistic regression analysis for early response recovery.

Parameters			CD (*n* = 47)				UC (*n* = 62)			
			Unadjusted		Adjusted		Unadjusted		Adjusted	
			OR (95% CI)	*P* value*	OR (95% CI)	*P* value*	OR (95% CI)	*P* value	OR (95% CI)	*P* value*
**Time to DE**		Weeks	1.01 (0.98-1.04)	.569	–	–	1.05 (1.00-1.10)	.068	3.01 (1.12-8.06)	.028
**Age**		Years	1.00 (0.95-1.06)	.999	–	–	0.99 (0.96-1.03)	.739	–	–
**Sex**		Male	1.58 (0.47-5.35)	.460	–	–	0.76 (0.26-2.26)	.625	–	–
**Race**		White	0 (0-inf)	.993	–	–	0 (0-Inf)	.992	–	–
**BMI at baseline**		kg/m^2^	0.96 (0.85-1.09)	.563	–	–	0.94 (0.83-1.08)	.398	–	–
**Disease Location (CD)/Disease extent (UC) at baseline**		Ileal related (CD)/Pancolitis (UC)	0.93 (0.27-3.14)	.905	–	–	0.66 (0.22-1.96)	.453	–	–
**Disease duration**		Years	1.00 (0.87-1.16)	.964	–	–	0.99 (0.91-1.08)	.875	–	–
**Prior use of biologics or JAKi**		Used	0.3 (0.04-2.01)	.214	0.03 (0.00-0.94)	.046	1.14 (0.31-4.21)	.848	–	–
**Oral corticosteroids at baseline**		Used	1.07 (0.32-3.57)	.917	–	–	1.58 (0.53-4.7)	.410	–	–
**ADA conversion before the time of LoR**		Positive	0.13 (0.03-0.69)	.016	0.04 (0.00-0.53)	.014	0.94 (0.32-2.78)	.915	–	–
**Clinical remission at week 10**		Remitter	1.73 (0.39-7.63)	.467	–	–	0.57 (0.17-1.93)	.370	–	–
**Albumin at the time of LoR**		g/L	0.91 (0.77-1.08)	.279	–	–	1.04 (0.91-1.19)	.557	–	–
**CDAI (CD)/MMS (UC)**	Baseline		1.01 (0.99-1.02)	.314	–	–	1.06 (0.64-1.74)	.826	–	–
	Week 10		1.00 (0.98-1.01)	.399	–	–	1.02 (0.65-1.61)	.926	–	–
	Time of LoR		1.00 (0.99-1.01)	.567	–	–	0.75 (0.45-1.23)	.248	–	–
	Change from Week 10 to LoR		1.01 (1.00-1.02)	.186	–	–	0.81 (0.54-6.76)	.296	0.48 (0.24-0.95)	.036
**CRP**	Baseline	nmol/L	1.00 (1.00-1.00)	.638	–	–	1.00 (1.00-1.01)	.464	–	–
	Week 10		1.03 (0.99-1.07)	.141	3.20 (1.29-7.96)	.012	1.00 (0.99-1.00)	.363	–	–
	Time of LoR		1.00 (1.00-1.00)	.469	–	–	1.00 (0.99-1.00)	.192	–	–
	Change from Week 10 to LoR		1.00 (1.00-1.00)	.689	–	–	1.00 (0.99-7.93)	.403	–	–
**Fecal calprotectin**	Baseline	mg/kg	1.00 (1.00-1.00)	.653	–	–	1.00 (1.00-1.00)	.044	3.40 (1.03-11.30)	.045
	Week 10		1.00 (1.00-1.00)	.584	–	–	1.00 (1.00-1.00)	.469	–	–
**Serum IFX levels**	Week 10	μg/ml	1.13 (1.01-1.27)	.040	1.25 (1.04-1.50)	.015	0.99 (0.90-1.08)	.790	–	–
	Time of LoR		1.03 (0.95-1.11)	.466	–	–	0.96 (0.89-1.03)	.273	–	–
	Change from LoR to Week 10		1.03 (0.96-1.11)	.399	–	–	1.04 (0.96-4.67)	.354	–	–

* Analyses were conducted descriptively and all *P*-values are nominal. For multivariable logistic regression, variables were transformed, including standardization and logarithmic transformations, as appropriate, to address issues such as non-normality and skewness, depending on the data distribution.

Abbreviations: ADA, antidrug antibodies; BMI, body mass index; CDAI, Crohn’s Disease Activity Index; CD, Crohn’s disease; CI, confidence interval; CRP, C-reactive protein; DE, dose escalation; IFX, infliximab; JAKi, Janus kinase inhibitor; LoR, loss of response; MMS, modified Mayo score; OR, odds ratio; UC, ulcerative colitis.

## Discussion

This post hoc analysis of LIBERTY-CD and LIBERTY-UC trials explored the effect of IFX SC DE from 120 mg to 240 mg Q2W as an effective strategy for achieving both rapid and sustained long-term response recovery in patients experiencing secondary loss of response.

Response recovery after DE was achieved in 85.1% (40/47) of patients with CD and 82.3% (51/62) of patients with UC. This was comparable to the rate observed in the REMSWITCH-LT study, where 82.1% (23/28) of patients with IBD achieved recapture of clinical remission after DE, although that study included a different DE regimen (120 mg weekly) and definition of clinical remission recapture.^21^ Furthermore, around two thirds of patients in this study achieved response recovery within 8 weeks of DE. This result aligns with previous studies on IFX IV DE, which reported comparable rates of 67-73% within 4-8 weeks of DE in CD^12^,^25^ and 68-90% within 4-12 weeks of DE in UC patients.^10^^,^^11^ This observation is notable given the pharmacokinetic differences between the IV and SC formulations,^14^^,^^26^ which may influence the timing and magnitude of clinical response. Additionally, even the late recovery group showed a statistically significant decrease in disease activity scores (CDAI/PMS) at their first visit after DE, suggesting that patients with an initial partial response to DE may still benefit from prolonged treatment to achieve full recovery.

Previous studies on IFX IV have demonstrated that a rapid recovery following DE is associated with better long-term outcomes, including reduced hospitalization and surgery.^10^^,^^11^^,^^27^ Consistent with these findings, the week 102 efficacy outcomes in this study showed that the early recovery group had better long-term outcomes compared to the late recovery group, suggesting that IFX SC may also be associated with favorable long-term outcomes when response recovery occurs early after DE. Limited benefits observed in the late recovery group, although one third of patients (three out of nine) achieved clinical remission but none achieved endoscopic response at week 102, may be due to insufficient follow-up period for these slowly responding patients or may indicate an ultimate failure of response recovery. Further studies are warranted to evaluate longer-term trajectories and outcomes of patients showing late recovery to better conclude benefits of DE in this population.

Prior to DE, serum IFX levels were below the concentration of 12-13 µg/mL shown in IFX SC therapeutic drug concentration studies as a requirement to sustain clinical remission during maintenance treatment.^24^^,^^28^ After DE, serum IFX levels significantly increased (22.1 µg/mL) in the early recovery group among patients with CD, but not in the late or non-recovery groups. This supports the role of IFX SC DE in resolving pharmacokinetic failure and suggests a potential role for drug optimization.

In patients with UC, a significant increase in serum IFX levels was observed in the early recovery group, also consistent with a pharmacokinetic failure that could be overcome with DE. However, in contrast to patients with CD, the non-recovery group showed a significant increase in serum IFX levels without clinical recovery, suggesting a mechanistic failure that could not be compensated by increasing drug levels.^29^^,^^30^ Reasons for mechanistic failures can include mechanistic differences (e.g., TNF-α-independent pathways^30^^,^^31^), serological characteristics,^32^ higher drug clearance,^33^^,^^34^ or genetic predispositions.^35^ Further research is needed to better understand these mechanisms.

Different predictive factors for early response recovery after DE were identified in patients with CD and UC. In patients with CD, variables related to pharmacokinetics and systemic inflammation, rather than local inflammation or disease course, were found to be significant. Higher serum IFX concentration at week 10 was associated with a greater likelihood of early recovery, while positive antidrug antibody conversion prior to loss of response was associated with a lower likelihood of early recovery, which aligns with the importance of the dose-exposure-response relationship from previous IFX IV studies. In addition, higher CRP levels at week 10 were associated with early recovery, suggesting that patients with elevated systemic inflammation despite induction therapy may respond more rapidly to increased IFX dosing. Additionally, prior exposure to biologic or JAKi was a negative predictor of early recovery, similar to previous observations with other standard-dose treatments.^36^^,^^37^

In patients with UC, variables related to disease course were found to be significant. A greater difference in MMS between week 10 and the week of loss of response was associated with a lower likelihood of early recovery, indicating that patients with greater disease worsening after induction may be less responsive to intensified dosing. Conversely, a marked worsening of MMS after initial response may reflect the involvement of non-TNF-α-mediated pathways,^30^^,^^31^^,^^38^ which are less responsive to IFX even at higher doses. In addition, a longer time to DE was associated with a higher likelihood of early recovery, implying that patients who maintained a response to IFX for prolonged periods prior to loss of response may have a disease phenotype more responsive to increased IFX therapy.

Given that the cost-effectiveness of a drug is a key determinant for use of medication and treatment sequencing in many countries, it may be important to assess the implications of DE on cost-effectiveness. While studies have suggested introduction of or switching to IFX SC may have a budget saving effect,^39^^,^^40^ these studies did not account for DE in the analyses, hence further studies that include DE in their scenarios are warranted. In addition, the relative cost-effectiveness of IFX SC DE compared to switching to another biologic would be helpful to determine treatment sequencing.

This study presents several important strengths. Most notably, it is the first study to explore the rapidity of response recovery following DE of IFX SC, data which can provide practical information for clinicians considering DE. Moreover, the use of high-quality, comprehensive data from phase 3 trials supports the robustness of the ­findings, with standardized assessments of disease activity and pha­rmacokinetics conducted across a well-defined patient population. The study limitations are its post hoc nature, modest sample size, and the lack of a corresponding cost-effectiveness analysis. As with any post hoc analysis, the strength of causal inferences is inherently limited. Additionally, the sample size was modest for identifying factors related to early response, leaving a possibility that our study was underpowered to identify meaningful factors. Regardless, these findings offer important insights to guide clinical decision-making for patients experiencing secondary loss of response to IFX SC.

In conclusion, this study demonstrates that DE of IFX SC is an effective strategy for restoring clinical response in patients with CD and UC following secondary loss of response. The majority of patients regained response within 8 weeks of escalation, and those in the early recovery group exhibited more favorable long-term outcomes, with sustained benefits observed through 102 weeks. These findings highlight the clinical utility of DE leading to early response recovery in most patients who lost response as a prerequisite for favorable long-term outcomes in IBD. The optimal use of individual dose adaptation needs to be explored in further prospective studies.

## Supplementary Material

izag017_Supplementary_Data

## Data Availability

A redacted study protocol has been published as part of the Supplementary Materials for an earlier publication.^4^ Individual participant data will not be shared.
