# Supplementary material for: Recovery of response and long-term outcomes following loss of response and dose escalation of subcutaneous infliximab: a post hoc analysis of the LIBERTY-CD & LIBERTY-UC trials
Source: Inflamm Bowel Dis. 2026 Mar 18;32(7):1235–47. doi: 10.1093/ibd/izag017 (PMC13337220; doi:10.1093/ibd/izag017)
Supplement: izag017_Supplementary_Data [file izag017_supplementary_data.docx]

# **SUPPLEMENT**

**Dubinsky MC et al.**

Recovery of Response and Long-term Outcomes Following Loss of Response and Dose Escalation of Subcutaneous Infliximab: A Post Hoc Analysis of the LIBERTY-CD & LIBERTY-UC Trials

**CONTENTS**

**1. Supplemental Methods 2**

Schedule of assessments for the LIBERTY-CD and LIBERTY-UC trials **2**

Corticosteroid tapering procedures for the LIBERTY-CD and LIBERTY-UC trials **3**

**2. Supplemental Figures** **4**

**Figure S1:** Patient disposition by response after dose escalation in LIBERTY-CD (A) and LIBERTY-UC (B) **4**

**Figure S2:** Time to dose escalation in patients with Crohn’s disease (A) and ulcerative colitis (B) **5**

**Figure S3:** Aalen–Johansen curves for time to response recovery after dose escalation in Crohn’s disease (A) and ulcerative colitis (B) **6**

**Figure S4:** C-reactive protein levels by response recovery status in Crohn’s disease (A) and ulcerative colitis (B) patients **7**

**3. Supplemental Tables** **8**

**Table S1**: Treatment-emergent adverse events during the maintenance phase before and after the dose escalation **8**

**Table S2:** Baseline characteristics by time to response recovery after dose escalation **10**

**Table S3:** Disease characteristics by time to response recovery after dose escalation **12**

**Table S4**: Patient characteristics before dose escalation at 6, 10, 14 weeks and at loss of response **14**

**Table S5**: Stool frequency and abdominal pain/rectal bleeding in Crohn’s disease and ulcerative colitis patients by time to response recovery following loss of response **17**

**Table S6**: Immunogenicity status by time to response recovery in Crohn’s disease and ulcerative colitis patients **18**

**Table S7**: Albumin level by time to response recovery in Crohn’s disease and ulcerative colitis patients **19**

# **1. Supplemental Methods**

## **Schedule of Assessments for the LIBERTY-CD and LIBERTY-UC trials**

**Efficacy Assessments**

In the LIBERTY-CD trial, Crohn’s Disease Activity Index (CDAI), abdominal pain (AP), and stool frequency (SF) were assessed at Screening and at weeks 2, 6, 10, 14, 22, 30, 38, 46, 54, 62, 70, 78, 86, 94, and 102. Patients completed the CDAI diary for at least 7 consecutive days prior to each assessment (including worst daily AP score and loose/watery SF), except when CDAI was measured on the same day as a colonoscopy. If the patient was planned to have bowel preparation for a colonoscopy procedure, the patient completed the CDAI diary for 7 consecutive days not to overlap with 3 days over the colonoscopy procedure. Colonoscopy with Simplified Endoscopic Activity Score for Crohn’s Disease (SES-CD) scoring was performed at Screening and at weeks 22, 54, and 102. All colonoscopy images were centrally evaluated by independent reviewers blinded to treatment assignment.

In the LIBERTY-UC trial, total and modified Mayo scores were obtained at Screening and at weeks 10, 22, 54, and 102. Partial Mayo scores were collected at Screening and at weeks 2, 6, 8, 10, 12, 14, 22, 30, 38, 46, 54, 62, 70, 78, 86, 94, and 102. Full colonoscopy could also be performed based on investigator’s discretion. If full colonoscopy was performed, it could replace flexible proctosigmoidoscopy. Flexible proctosigmoidoscopy (or full colonoscopy) for endoscopic subscore assessment was performed within 14 days prior to the Mayo score assessment. Flexible proctosigmoidoscopy (or full colonoscopy) could be performed whenever needed based on investigator’s discretion including determination of loss of response. Endoscopic subscore by flexible proctosigmoidoscopy (or full colonoscopy) was evaluated at the central level by an independent reviewer blinded to treatment allocation to confirm eligibility, determine loss of response, and for reporting purposes. The local endoscopic subscore was considered during evaluation of the endoscopic subscore at the central level. Patients completed a 7-day Mayo diary before each assessment; the most recent 3 days (not necessarily consecutive) were used for score calculation. When the assessment coincided with a flexible sigmoidoscopy or colonoscopy, days overlapping the procedure (the day before through the day after) were excluded. Histologic assessment using the Robarts Histopathology Index (RHI) was performed at Screening and at weeks 10, 22, 54, and 102, and centrally reviewed by independent, treatment-blinded readers.

**Safety, Pharmacokinetic, and Other Assessments**

Adverse events were assessed from the date the patient signed the informed consent form until the last assessment date or end of study (EOS) visit. Where an adverse drug reaction (ADR) (i.e., related to study drug) was ongoing at the EOS visit, the ADR was followed up until one of the following: resolution or improvement from baseline, relationship reassessed as unrelated, confirmation from the investigator that no further improvement could be expected, end of collection of clinical or safety data, or final database closure. Adverse events of special interest (i.e., infusion-related reaction/systemic injection reaction, infection, delayed hypersensitivity, localized injection site reaction, and malignancy) were to be closely monitored.

Immunogenicity was assessed as follows: Serum samples for anti-drug antibody (ADA) testing were collected pre-dose at weeks 0, 10, 14, 22, 30, 38, 46, 54, 62, 70, 78, 86, 94, and 102. Additional samples were collected if delayed hypersensitivity or serum sickness was suspected. All analyses were performed at a central laboratory.

Pharmacokinetics and pharmacodynamics were assessed as follows: Blood samples for pharmacokinetic analysis were collected pre-dose at weeks 0, 2, 6, 10, 14, 22, 30, 38, 46, 54, 62, 70, 78, 86, 94, and 102. C-reactive protein (CRP) was measured at weeks 0, 2, 6, 10, 14, 22, 30, 38, 46, 54, 62, 70, 78, 86, 94, and 102. Fecal calprotectin was assessed at weeks 0, 10, 22, 54, 70, 86, and 102.

To evaluate quality of life, the Short Inflammatory Bowel Disease Questionnaire (SIBDQ) was administered at weeks 0, 2, 6, 10, 14, 22, 30, 38, 46, 54, 70, 86, and 102.

Data from unscheduled visits were not included in visit-based summaries unless otherwise specified.

## **Corticosteroid tapering procedures for the LIBERTY-CD and LIBERTY-UC trials**

Oral corticosteroids at the equivalent dose of 20 mg/day or less of prednisone were allowed if the patient had received a stable dose for at least 2 weeks prior to the first administration of the study drug (Day 0). For patients receiving corticosteroids at the first administration of the study drug (Day 0), corticosteroid treatment was kept up to week 10 at the same dose level. After week 10, the dose was tapered, and the following tapering regimen was recommended: a tapering rate of 2.5 mg/week was recommended, with a maximum rate of 5 mg/week if current corticosteroids dose was >10 mg/day equivalent dose of prednisone. In case of corticosteroid dose of ≤10 mg/day as equivalent dose of prednisone, tapering rate was recommended as 2.5 mg/week.

Oral budesonide at the dose of 9 mg/day or less was allowed if the patient had received a stable dose for at least 2 weeks prior to the first administration of the study drug (Day 0). For patients receiving budesonide at the first administration of the study drug (Day 0), budesonide treatment was kept up to week 10 at the same dose. After week 10, the dose was tapered, and tapering rate of 3 mg every 2 weeks was recommended. The investigators could also follow local clinical practice for the tapering regimen.

# **2. Supplemental Figures**

## **Figure S1:** Patient disposition by response after dose escalation in LIBERTY-CD (**A**) and LIBERTY-UC (**B**)


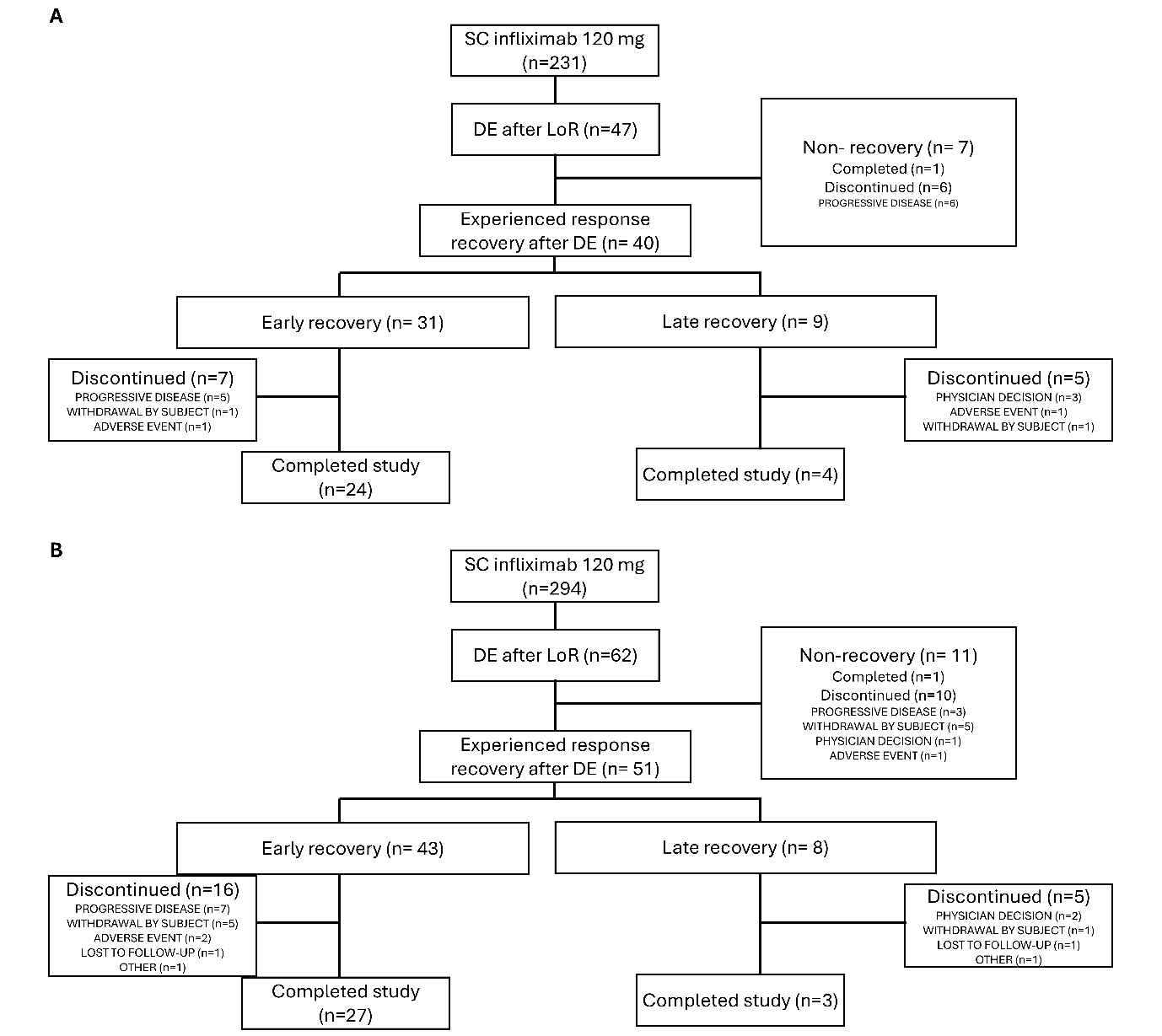


Patients were classified into three groups based on time to response recovery: the early recovery group (patients who achieved response recovery within 8 weeks following DE), the late recovery group (patients who achieved response recovery after 8 weeks), and the non-recovery group (patients who were censored before achieving recovery).

Abbreviations: CD, Crohn’s disease; DE, dose escalation; LoR, loss of response; SC, subcutaneous; UC, ulcerative colitis.

**Figure S2:** Time to dose escalation in patients with Crohn’s disease (A) and ulcerative colitis (B)


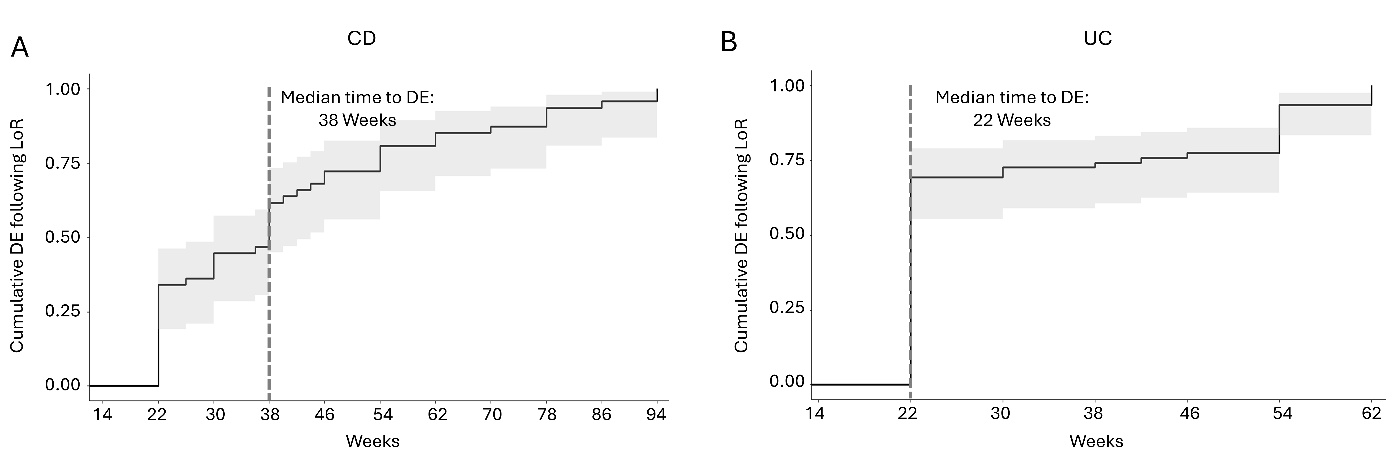


Abbreviations: CD, Crohn’s disease; DE, dose escalation; LoR, loss of response; UC, ulcerative colitis.

## **Figure S3:** Aalen–Johansen curves for time to response recovery after dose escalation in Crohn’s disease (A) and ulcerative colitis (B)

**
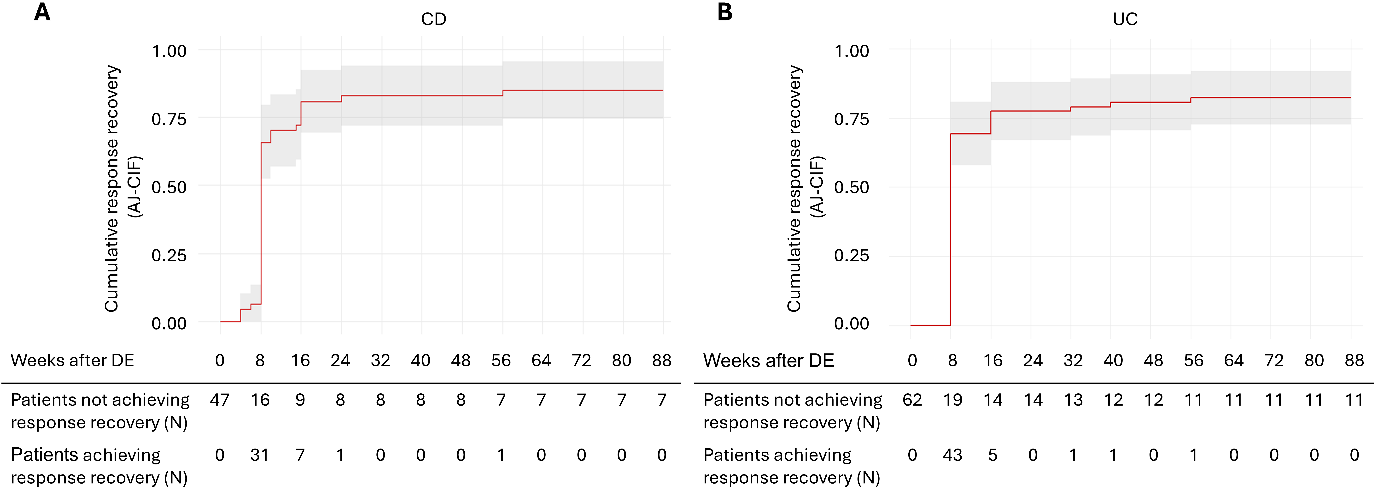
**

Patients were tested for their CDAI score or PMS on an 8-weekly basis. However, the timing of the relative visit may vary for some patients due to unscheduled DE visits. In order to conduct the time-to-event type analysis in patients with UC, the analysis was conducted using the PMS with a short efficacy measurement interval (8-weeks term). Response recovery for CD: CDAI-100 from loss of response; Response recovery for UC: Decrease in PMS from loss of response of at least 2 points, with an accompanying decrease in the RB of at least 1 point, or an absolute RB of 0 or 1 point.

Abbreviations: AJ-CIF, Aalen–Johansen cumulative incidence function; CDAI, Crohn's Disease Activity Index; CD, Crohn’s disease; DE, dose escalation; PMS, partial Mayo score; RB, rectal bleeding subscore; UC, ulcerative colitis.

## **Figure S4:** C-reactive protein levels by response recovery status in Crohn’s disease (A) and ulcerative colitis (B) patients


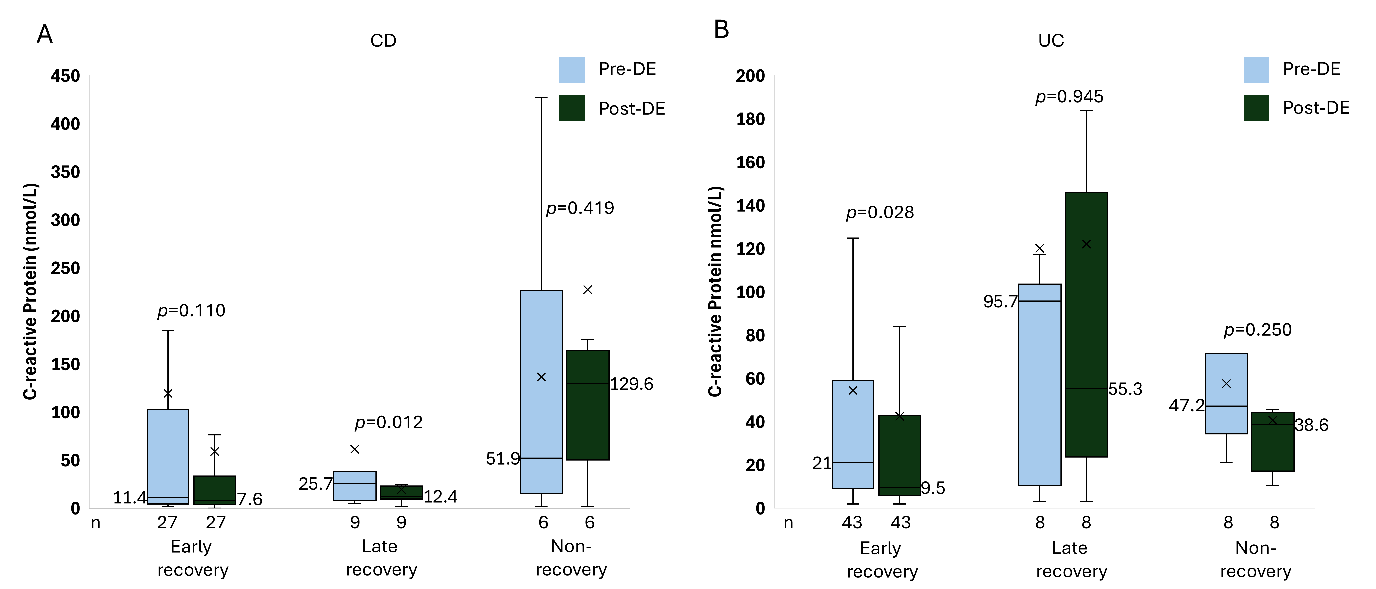


The box plot displays the median (line within the box), mean (×), IQR (box from Q1 to Q3), and the minimum and maximum values excluding outliers (whiskers). Patients were classified into three groups based on time to response recovery: the early recovery group (patients who achieved response recovery within 8 weeks following DE), the late recovery group (patients who achieved response recovery after 8 weeks), and the non-recovery group (patients who were censored before achieving recovery). Pre-DE refers to the time of LoR, which is the week when patients experience a LoR. Post-DE refers to the first visit after DE, representing the time points for assessing CDAI/PMS following DE, including not only scheduled visits but also unscheduled visits and end of the study. Analyses were conducted on paired samples descriptively, and the nominal p-values were calculated by comparing Pre-DE and Post-DE data using the paired Wilcoxon signed-rank test.

Abbreviations: CDAI, Crohn’s Disease Activity Index; CD, Crohn’s disease; DE, dose escalation; IQR, interquartile range; LoR, loss of response; PMS, partial Mayo score; UC, ulcerative colitis.

# **3. Supplemental Tables**

## **Table S1:** Treatment-emergent adverse events during the maintenance phase before and after the dose escalation

| **Variable, n (%)** | **CD (n=47)** | | **UC (n=62)** | |
| --- | --- | --- | --- | --- |
|  | **Before DE** | **After DE** | **Before DE** | **After DE** |
| **Patients with ≥1 TEAE** | 31 (66.0) | 28 (59.6) | 35 (56.5) | 39 (63.0) |
| **Study drug–related** | 14 (29.8) | 9 (19.1) | 13 (21.0) | 13 (21.0) |
| **Study drug–unrelated** | 28 (59.6) | 25 (53.2) | 32 (51.6) | 35 (56.5) |
| **Patients with ≥1 TESAE** | 2 (4.3) | 4 (8.6) | 1 (1.7) | 6 (9.7) |
| **Study drug–related** | 0 (0) | 0 (0) | 0 (0) | 0 (0) |
| **Study drug–unrelated** | 2 (4.3) | 4 (8.6) | 1 (1.7) | 6 (9.7) |
| **Patients with ≥1 TEAE classified as ISR** | 2 (4.3) | 4 (8.6) | 4 (6.5) | 1 (1.7) |
| **Study drug–related** | 2 (4.3) | 4 (8.6) | 4 (6.5) | 1 (1.7) |
| **Study drug–unrelated** | 0 (0) | 0 (0) | 0 (0) | 0 (0) |
| **Patients with ≥1 TEAE classified as infection** | 14 (29.8) | 19 (40.5) | 13 (21.0) | 19 (30.7) |
| **Study drug–related** | 2 (4.3) | 2 (4.3) | 4 (6.5) | 4 (6.5) |
| **Study drug–unrelated** | 14 (29.8) | 19 (40.5) | 11 (17.7) | 17 (27.4) |
| **Patients with ≥1 TEAE classified as malignancy** | 0 (0) | 0 (0) | 0 (0) | 0 (0) |
| **Study drug–related** | 0 (0) | 0 (0) | 0 (0) | 0 (0) |
| **Study drug–unrelated** | 0 (0) | 0 (0) | 0 (0) | 0 (0) |
| **Patients with ≥1 TEAE leading to death** | 0 (0) | 0 (0) | 0 (0) | 0 (0) |
| **Study drug–related** | 0 (0) | 0 (0) | 0 (0) | 0 (0) |
| **Study drug–unrelated** | 0 (0) | 0 (0) | 0 (0) | 0 (0) |

Before DE indicates the period after induction when patients were treated with 120 mg Q2W prior to dose escalation. After DE indicates the period during which patients received 240 mg Q2W.

Abbreviations**:** CD, Crohn’s disease; DE, dose escalation; ISR, injection-site reaction; TEAE, treatment-emergent adverse event; TESAE, treatment-emergent serious adverse event; UC, ulcerative colitis.

## **Table S2:** Patients characteristics by time to response recovery after dose escalation

| **Baseline characteristics** |  | **CD** | | | | **UC** | | | |
| --- | --- | --- | --- | --- | --- | --- | --- | --- | --- |
|  |  | **Early**  **recovery**  **(N=31)** | **Late**  **recovery**  **(N=9)** | **Non-**  **recovery**  **(N=7)** | **P value** | **Early**  **recovery**  **(N=43)** | **Late**  **recovery**  **(N=8)** | **Non-**  **recovery**  **(N=11)** | **P value** |
| **Time to DE** | Week | 38.0  [22.0-54.0] | 22.0  [22.0-38.0] | 36.0  [26.0-49.0] | 0.412 | 22.0  [22.0-54.0] | 22.0  [22.0-22.0] | 22.0  [22.0-22.0] | 0.164 |
| **Age** | Year | 35.0  [28.5-42.5] | 36.0  [30.0-38.0] | 31.0  [29.0-37.0] | 0.794 | 36.0  [31.0-44.5] | 46.0  [30.0-59.5] | 31.0  [27.5-48.5] | 0.549 |
| **Sex, n (%)** | Male | 19 (61.3) | 5 (55.6) | 3 (42.9) | 0.667 | 22 (51.2) | 6 (75.0) | 5 (45.5) | 0.394 |
| **Race, n (%)** | White | 24 (77.4) | 8 (88.9) | 7 (100.0) | 0.528 | 42 (97.7) | 8 (100.0) | 11 (100.0) | 0.799 |
| **BMI at baseline** | kg/m^2^ | 24.0 ± 4.5 | 26.5 ± 5.0 | 22.7 ± 5.9 | 0.244 | 23.5 ± 4.0 | 26.3 ± 4.7 | 23.0 ± 3.2 | 0.142 |
| **Disease location (CD) /**  **Disease extent (UC) at**  **baseline, n (%)** |  |  |  |  | 0.655 |  |  |  | 0.633 |
| **Colonic CD / Pancolitis** | | 18 (58.1) | 4 (44.4) | 5 (71.4) |  | 16 (37.2) | 4 (50.0) | 5 (45.5) |  |
| **Ileal-colonic CD /**  **Left-sided colitis** | | 10 (32.3) | 3 (33.3) | 2 (28.6) |  | 22 (51.2) | 2 (25.0) | 4 (36.4) |  |
| **Ileal CD / Ulcerative**  **proctosigmoiditis** | | 3 (9.7) | 2 (22.2) | 0 (0.0) |  | 5 (11.6) | 2 (25.0) | 2 (18.2) |  |
| **Disease duration** | Year | 2.8  [1.0- 6.3] | 3.8  [1.5-10.1] | 2.4  [1.8- 4.5] | 0.848 | 6.7  [3.0-10.9] | 7.4  [3.9-10.6] | 7.7  [5.4-9.8] | 0.883 |
| **Prior use of biologics or JAKi, n (%)** | Used | 2 (6.5) | 2 (22.2) | 1 (14.3) | 0.379 | 10 (23.3) | 2 (25.0) | 2 (18.2) | 0.923 |
| **Immunosuppressant (AZA, 6-MP, MTX) at baseline, n (%)** | Used | 16 (51.6) | 1 (11.1) | 3 (42.9) | 0.096 | 11 (25.6) | 3 (37.5) | 1 (9.1) | 0.335 |
| **Oral corticosteroids at week 0, n (%)** | Used | 16 (51.6) | 4 (44.4) | 4 (57.1) | 0.876 | 23 (53.5) | 2 (25.0) | 6 (54.5) | 0.317 |
| **Oral corticosteroids at the time of DE, n (%)** | Used | 5 (16.1) | 0 | 1 (14.3) | 0.439 | 7 (16.3) | 0 | 1 (9.1) | 0.414 |
| **Clinical remission at week 10, n (%)** | Remitter | 26 (83.9) | 6 (66.7) | 6 (85.7) | 0.482 | 9 (20.9) | 3 (37.5) | 3 (27.3) | 0.583 |
| **Albumin at the time of LoR** | g/L | 45.0  [42.0-47.5] | 46.0  [44.0-48.0] | 43.5  [42.0-46.0] | 0.284 | 43.9 ± 3.8 | 43.1 ± 4.5 | 43.0 ± 4.8 | 0.756 |
| **ADA conversion before the**  **time of LoR, n (%)** | Positive | 15 (48.4) | 8 (88.9) | 6 (85.7) | **0.033** | 22 (51.2) | 4 (50.0) | 6 (54.5) | 0.975 |

These data were generated using the mytable function (method 3) from the moonBook package in R (version 4.3.2). Variables following a normal distribution, as determined by the Shapiro-Wilk test, are presented as mean (± SD), while non-normally distributed variables are presented as median [IQR]. Analyses were conducted descriptively and all P values are nominal.

Abbreviation**:** ADA, anti-drug antibody; AZA, azathioprine; BMI, body mass index; CD, Crohn’s disease; DE, dose escalation; IQR, interquartile range; 6-MP, 6-mercaptopurine; MTX, methotrexate; JAKi, Janus kinase inhibitor; LoR, loss of response; SD, standard deviation; UC, ulcerative colitis.

## **Table S3:** Disease characteristics by time to response recovery after dose escalation

| **Disease**  **Characteristics** | **Time** | **CD** | | | | **UC** | | | |
| --- | --- | --- | --- | --- | --- | --- | --- | --- | --- |
|  |  | **Early**  **recovery**  **(N=31)** | **Late**  **recovery**  **(N=9)** | **Non-**  **recovery**  **(N=7)** | **P value** | **Early**  **recovery**  **(N=43)** | **Late**  **recovery**  **(N=8)** | **Non-**  **recovery**  **(N=11)** | **P value** |
| **CDAI (CD)/**  **MMS (UC)^†^** | Baseline | 328.7  [282.2-369.9] | 301.8  [240.5-383.0] | 303.2  [269.1-353.1] | 0.506 | 7.0  [6.0-8.0] | 7.0  [6.5-7.0] | 7.0  [5.5-7.5] | 0.924 |
|  | Week 10 | 89.8 ± 53.3 | 128.3 ± 74.0 | 74.4 ± 40.3 | 0.124 | 3.0  [2.0-3.5] | 2.5  [1.0-4.0] | 3.0  [2.0-3.5] | 0.838 |
|  | Week 22 | 128.9 ± 93.7 | 198.8 ± 92.0 | 137.7 ± 139.5 | 0.196 | 5.0  [2.0-7.0] | 5.5  [5.0-6.5] | 7.0  [6.5-7.0] | **0.035** |
|  | Time of LoR | 267.6  [236.3-313.6] | 251.0  [237.4-317.6] | 234.8  [227.4-276.2] | 0.461 | 6.0  [5.0-7.0] | 5.5  [5.0-6.5] | 7.0  [7.0-8.0] | **0.005** |
| **SES-CD (CD)^‡^/**  **MES (UC)†** | Baseline | 12.6 ± 5.7 | 9.3 ± 6.3 | 15.7 ± 8.2 | 0.133 | 3.0  [2.0-3.0] | 3.0  [2.5-3.0] | 3.0  [2.0-3.0] | 0.866 |
|  | Week 10 | N/A | N/A | N/A | N/A | 2.0  [1.0-2.0] | 1.5  [1.0-3.0] | 2.0  [1.0-2.0] | 0.804 |
|  | Week 22 | 6.0 ± 7.1 | 4.6 ± 4.5 | 6.6 ± 8.9 | 0.818 | 2.0  [2.0-3.0] | 2.5  [2.0-3.0] | 3.0  [3.0-3.0] | 0.149 |
|  | Time of LoR | N/A | N/A | N/A | N/A | 3.0  [2.0-3.0] | 2.5  [2.0-3.0] | 3.0  [3.0-3.0] | 0.084 |
| **C-reactive protein**  **(nmol/L)** | Baseline | 67.6  [27.1-135.7] | 13.3  [12.4-27.6] | 45.7  [21.9-65.7] | 0.075 | 31.4  [12.4-101.0] | 56.6  [32.9-88.1] | 7.6  [3.3-28.5] | **0.031** |
|  | Week 10 | 11.4  [4.8-37.2] | 8.6  [5.7-11.4] | 12.4  [2.4-23.8] | 0.611 | 12.4  [3.8-36.2] | 70.5  [7.6-129.5] | 9.5  [4.3-29.0] | 0.281 |
|  | Week 22 | 10.0  [5.7-35.2] | 23.8  [11.4-28.6] | 35.2  [11.0-129.1] | 0.585 | 14.3  [5.7-46.2] | 52.4  [8.6-108.0] | 34.3  [13.3-64.8] | 0.525 |
|  | Time of LoR | 11.4  [4.3-102.8] | 25.7 [7.6-38.1] | 51.9  [14.3-272.4] | 0.766 | 21.0  [9.1-59.1] | 95.7  [9.5-108.0] | 47.2  [34.3-71.4] | 0.160 |
| **Fecal calprotectin^§^**  **(mg/kg)** | Baseline | 1117.0  [654.0-4477.0] | 1239.0  [1134.0-2009.0] | 3614.0  [775.5-5088.5] | 0.772 | 2181.5  [881.0-5915.0] | 1681.0  [981.0-1745.5] | 839.0  [395.5-2781.0] | 0.075 |
|  | Week 10 | 167.0  [31.5-695.0] | 665.5  [80.0-1253.0] | 97.0  [48.0-184.0] | 0.358 | 554.0  [100.0-1860.0] | 644.0  [266.0-1337.0] | 529.0  [43.5-817.0] | 0.668 |
|  | Week 22 | 110.5  [31.0-526.0] | 558.5  [136.0-1334.0] | 189.0  [65.0-901.0] | 0.245 | 677.0  [100.0-2034.0] | 623.5  [189.5-1197.5] | 1253.0  [369.0-1977.0] | 0.649 |
| **Serum IFX levels**  **(μg/ml)** | Week 10 | 13.6 ± 6.4 | 9.2 ± 5.4 | 10.1 ± 4.2 | 0.103 | 11.2 ± 6.3 | 11.5 ± 5.5 | 11.8 ± 6.1 | 0.962 |
|  | Week 22 | 12.7 ± 8.3 | 11.3 ± 9.7 | 8.1 ± 8.4 | 0.430 | 11.8 ± 7.1 | 10.6 ± 6.3 | 10.9 ± 6.9 | 0.880 |
|  | Time of LoR | 8.1  [1.7-16.2] | 11.1  [0.1-17.4] | 0.1  [0.1-2.3] | 0.138 | 9.5 ± 5.9 | 10.4 ± 6.6 | 9.7 ± 5.9 | 0.915 |

These data were generated using the mytable function (method 3) from the moonBook package in R (version 4.3.2). Variables following a normal distribution by the Shapiro-Wilk test, are presented as mean (± SD), while non-normally distributed variables are presented as median [IQR]. Analyses were conducted descriptively and all P values are nominal.

^†^MMS was not tested on an 8-week basis; however, since the LoR criteria were based on MMS, all patients had an MMS score at the time of LoR.

^‡^SES-CD was only tested at weeks 0, 22, 54 and 102.

^§^Since fecal calprotectin was not tested on an 8-week basis, its value at the time of LoR was not included.

Abbreviations: CDAI, Crohn's Disease Activity Index; CD, Crohn’s disease; IFX, infliximab; IQR, interquartile range; LoR, loss of response; MES, Mayo endoscopic score; MMS, modified Mayo score; N/A, not applicable; SD, standard deviation; SES-CD, Simplified endoscopic score for Crohn's disease; UC, ulcerative colitis.

**Table S4:** Patient characteristics before dose escalation at 6, 10, 14 weeks and at loss of response

|  | **CD** | | | | **UC** | | | |
| --- | --- | --- | --- | --- | --- | --- | --- | --- |
| **Week 6 (last induction week)** | **Early Recovery**  **(N=31)** | **Late Recovery**  **(N=9)** | **Non-Recovery**  **(N=7)** | **P value** | **Early Recovery**  **(N=43)** | **Late Recovery**  **(N=8)** | **Non-Recovery**  **(N=11)** | **P value** |
| Albumin (g/L) | 45.1 ± 4.4 | 45.8 ± 2.7 | 46.1 ± 2.9 | 0.798 | 44.8 ± 3.7 | 43.0 ± 3.8 | 46.0 ± 3.5 | 0.223 |
| CDAI (CD) /PMS (UC) | 136.0 ± 97.2 | 183.4 ± 70.2 | 133.0 ± 67.6 | 0.357 | 2.6 ± 2.1 | 2.4 ± 2.1 | 2.6 ± 1.8 | 0.945 |
| SF | 1.9 ± 1.6 | 1.9 ± 1.5 | 2.0 ± 2.2 | 0.947 | 0.9 ± 0.9 | 1.2 ± 1.3 | 1.4 ± 0.9 | 0.322 |
| AP (CD) / RB (UC) | 0.9 ± 0.7 | 1.3 ± 0.7 | 0.8 ± 0.8 | 0.257 | 0.6 ± 0.7 | 0.4 ± 0.7 | 0.2 ± 0.4 | 0.212 |
| CRP | 24.5 ± 34.6 | 13.4 ± 11.7 | 20.4 ± 21.1 | 0.912 | 30.6 ± 66.5 | 112.5 ± 170.9 | 17.4 ± 21.8 | 0.728 |
| SIBDQ | 51.7 ± 10.7 | 45.0 ± 5.7 | 47.0 ± 10.6 | 0.165 | 50.9 ± 11.3 | 52.2 ± 13.2 | 46.5 ± 8.9 | 0.360 |
| Serum IFX levels (μg/mL) | 14.5 ± 8.0 | 11.1 ± 4.0 | 9.6 ± 5.1 | 0.174 | 11.3 ± 7.9 | 12.3 ± 5.8 | 11.6 ± 5.0 | 0.942 |
| **Week 10 (end of induction)** |  |  |  |  |  |  |  |  |
| Albumin (g/L) | 45.2 ± 4.1 | 46.7 ± 3.8 | 45.7 ± 2.4 | 0.598 | 45.1 ± 3.5 | 43.5 ± 3.7 | 45.4 ± 3.9 | 0.469 |
| CDAI (CD) /PMS (UC) | 89.8 ± 53.3 | 128.3 ± 74.0 | 74.4 ± 40.3 | 0.124 | 1.7 ± 1.1 | 1.2 ± 0.9 | 2.0 ± 1.7 | 0.485 |
| SF | 1.0 ± 0.9 | 1.6 ± 1.0 | 0.7 ± 0.8 | 0.149 | 0.6 ± 0.7 | 0.6 ± 0.7 | 1.1 ± 0.9 | 0.285 |
| AP (CD) / RB (UC) | 0.6 ± 0.6 | 0.8 ± 0.4 | 0.6 ± 0.6 | 0.428 | 0.2 ± 0.4 | 0.1 ± 0.4 | 0.1 ± 0.3 | 0.502 |
| CRP | 33.3 ± 64.6 | 11.1 ± 9.1 | 13.7 ± 12.4 | 0.611 | 35.2 ± 71.2 | 98.1 ± 121.1 | 23.4 ± 31.9 | 0.281 |
| SIBDQ | 55.6 ± 9.0 | 45.2 ± 10.6 | 51.7 ± 11.8 | 0.024 | 56.5 ± 7.7 | 58.2 ± 8.3 | 47.2 ± 12.2 | 0.006 |
| Pain intensity (mm) | 14.7 ± 21.3 | 13.1 ± 12.6 | 28.1 ± 26.7 | 0.231 | 8.8 ± 7.7 | 6.9 ± 7.0 | 16.0 ± 24.7 | 0.541 |
| Serum IFX levels (μg/mL) | 13.6 ± 6.4 | 9.2 ± 5.4 | 10.1 ± 4.2 | 0.103 | 11.2 ± 6.3 | 11.5 ± 5.5 | 11.8 ± 6.1 | 0.962 |
| ADA Positive, n (%) | 1 (3.2) | 0 (0.0) | 2 (28.6) | 0.032 | 6 (14.0) | 0 (0.0) | 3 (27.3) | 0.245 |
| **Week 14 (right after SC switching)** |  |  |  |  |  |  |  |  |
| Albumin (g/L) | 45.4 ± 4.9 | 46.0 ± 3.6 | 45.6 ± 4.0 | 0.939 | 44.9 ± 3.4 | 43.6 ± 5.1 | 45.6 ± 2.6 | 0.472 |
| CDAI (CD) / PMS (UC) | 100.3 ± 55.7 | 110.0 ± 62.6 | 114.2 ± 97.4 | 0.929 | 1.9 ± 1.6 | 2.2 ± 1.9 | 2.8 ± 2.2 | 0.496 |
| SF | 0.9 ± 0.9 | 1.1 ± 1.0 | 1.9 ± 2.7 | 0.645 | 0.8 ± 0.8 | 1.4 ± 1.3 | 1.6 ± 1.3 | 0.119 |
| AP (CD) / RB (UC) | 0.6 ± 0.5 | 0.7 ± 0.7 | 0.8 ± 0.9 | 0.955 | 0.3 ± 0.7 | 0.2 ± 0.7 | 0.2 ± 0.4 | 0.704 |
| CRP | 34.4 ± 83.5 | 13.2 ± 11.4 | 20.3 ± 23.1 | 0.724 | 23.3 ± 30.3 | 137.1 ± 179.3 | 30.8 ± 45.0 | 0.379 |
| SIBDQ | 56.4 ± 8.3 | 48.3 ± 8.6 | 54.3 ± 9.8 | 0.054 | 53.8 ± 11.0 | 53.2 ± 10.8 | 42.7 ± 14.5 | 0.067 |
| Pain intensity (mm) | 9.8 ± 12.3 | 11.8 ± 11.0 | 22.4 ± 29.3 | 0.739 | 9.3 ± 12.2 | 10.1 ± 13.8 | 11.7 ± 21.1 | 0.502 |
| Serum IFX levels (μg/mL) | 14.4 ± 5.5 | 12.0 ± 7.6 | 13.9 ± 5.3 | 0.762 | 13.2 ± 6.0 | 12.9 ± 6.3 | 13.6 ± 5.9 | 0.972 |
| ADA Positive, n (%) | 6 (19.4) | 3 (33.3) | 2 (28.6) | 0.643 | 12 (27.9) | 1 (12.5) | 6 (54.5) | 0.114 |
| **Time of LoR** |  |  |  |  |  |  |  |  |
| Albumin (g/L) | 44.7 ± 4.5 | 46.7 ± 3.1 | 43.8 ± 2.9 | 0.284 | 43.9 ± 3.8 | 43.1 ± 4.5 | 43.0 ± 4.8 | 0.756 |
| CDAI (CD) / PMS (UC) | 283.9 ± 67.4 | 282.3 ± 58.2 | 260.8 ± 48.8 | 0.461 | 5.8 ± 1.3 | 4.9 ± 1.0 | 6.3 ± 0.6 | 0.030 |
| SF | 4.8 ± 3.4 | 4.2 ± 1.7 | 4.3 ± 3.0 | 0.869 | 2.1 ± 0.7 | 2.4 ± 0.9 | 2.9 ± 0.3 | 0.003 |
| AP (CD) / RB (UC) | 2.0 ± 0.4 | 2.1 ± 0.5 | 1.8 ± 0.4 | 0.352 | 1.6 ± 0.7 | 0.9 ± 0.8 | 1.5 ± 0.8 | 0.069 |
| CRP | 119.3 ± 218.5 | 61.3 ± 105.8 | 136.5 ± 174.6 | 0.766 | 54.4 ± 100.7 | 120.1 ± 172.8 | 89.8 ± 126.2 | 0.160 |
| SIBDQ | 39.2 ± 13.9 | 38.5 ± 8.9 | 40.2 ± 11.4 | 0.928 | 43.2 ± 11.2 | 37.5 ± 15.8 | 27.5 ± 7.8 | 0.001 |
| Pain intensity (mm) | 10.8 ± 15.8 | 14.2 ± 9.7 | 39.4 ± 42.3 | 0.272 | 16.4 ± 20.4 | 8.9 ± 11.3 | 10.6 ± 18.3 | 0.113 |
| Serum IFX levels (μg/mL) | 9.4 ± 7.6 | 10.5 ± 10.7 | 3.5 ± 7.2 | 0.138 | 9.5 ± 5.9 | 10.5 ± 6.6 | 9.7 ± 5.9 | 0.915 |
| ADA Positive, n (%) | 13 (43.3) | 7 (77.8) | 6 (85.7) | 0.045 | 19 (45.2) | 4 (50.0) | 6 (60.0) | 0.699 |

Patients were classified into three groups based on time to response recovery: the early recovery group (patients who achieved response recovery within 8 weeks following DE), the late recovery group (patients who achieved response recovery after 8 weeks), and the non-recovery group (patients who were censored before achieving recovery). All values are presented as mean ($\pm$SD) for continuous variables and n (%) for dichotomous variables. P-values were generated using the mytable function (method 3) in the moonBook package in R. Analyses were conducted descriptively and all P values are nominal. Stool frequency (SF) for CD refers to the average daily SF score, while for UC, it represents the Mayo SF subscore. Abdominal pain (AP) for CD denotes the average daily AP score, whereas rectal bleeding (RB) for UC corresponds to the Mayo RB subscore.

Abbreviations: ADA, antidrug antibody; AP, abdominal pain; CD, Crohn’s disease; CDAI, Crohn's Disease Activity Index; CRP, C-reactive protein; DE, dose escalation; IFX, infliximab; LoR, loss of response; PMS, partial Mayo score; RB, rectal bleeding; SC, subcutaneous; SD, standard deviation; SF, stool frequency; SIBDQ, Short Inflammatory Bowel Disease Questionnaire; UC, ulcerative colitis.

## **Table S5:** Stool frequency and abdominal pain/rectal bleeding in patients with Crohn’s disease and ulcerative colitis by time to response recovery following loss of response

| **Patient Reported**  **Outcomes** | **CD** | | | | **UC** | | | | |
| --- | --- | --- | --- | --- | --- | --- | --- | --- | --- |
|  | **Early Recovery**  **(N=31)** | **Late Recovery**  **(N=9)** | **Non-Recovery**  **(N=7)** | **P value** | **Early Recovery**  **(N=43)** | **Late Recovery**  **(N=8)** | **Non-Recovery**  **(N=8)** | **P value** |  |
| **Stool frequency,** Mean (±SD) | | | | | | | | | |
| Pre-DE | 4.8 ± 3.4 | 4.2 ± 1.7 | 4.3 ± 3.0 | 0.869 | 1.6 ± 0.7 | 0.9 ± 0.8 | 1.2 ± 0.7 | 0.046 |  |
| Post-DE | 1.2 ± 1.8 | 1.5 ± 1.3 | 5.3 ± 4.8 | 0.012 | 0.1 ± 0.3 | 0.8 ± 0.9 | 1.2 ± 0.9 | <0.001 |  |
| Change from Pre- to Post-DE | -3.6 ± 2.2 | -2.7 ± 2.1 | 0.9 ± 3.4 | <0.001 | -1.5 ± 0.7 | -0.1 ± 0.4 | 0.0 ± 0.8 | <0.001 |  |
| **Abdominal pain (CD) / Rectal bleeding (UC),** Mean (±SD) | | | | | | | | | |
| Pre-DE | 2.0 ± 0.4 | 2.1 ± 0.5 | 1.8 ± 0.4 | 0.352 | 2.1 ± 0.7 | 2.4 ± 0.9 | 2.9 ± 0.4 | 0.015 |  |
| Post-DE | 0.5 ± 0.6 | 1.4 ± 0.8 | 1.4 ± 0.9 | 0.001 | 0.8 ± 0.7 | 2.1 ± 0.8 | 2.8 ± 0.5 | <0.001 |  |
| Change from Pre- to Post-DE | -1.5 ± 0.6 | -0.7 ± 0.6 | -0.4 ± 0.9 | <0.001 | -1.3 ± 0.9 | -0.2 ± 0.5 | -0.1 ± 0.4 | <0.001 |  |

Pre-DE refers to the time of LoR, which is the week when patients experience a LoR. Post-DE refers to the first visit after DE, representing the time points for assessing CDAI/PMS following DE, including not only scheduled visits but also unscheduled visits and end of study visits. Stool frequency (SF) for CD refers to the average daily SF score, while for UC, it represents the Mayo SF subscore. Abdominal pain (AP) for CD denotes the average daily AP score, whereas rectal bleeding (RB) for UC corresponds to the Mayo RB subscore. All values are presented as mean (±SD) for continuous variables and n (%) for dichotomous variables. Patients were classified into three groups based on time to response recovery: the early recovery group (patients who achieved response recovery within 8 weeks following DE), the late recovery group (patients who achieved response recovery after 8 weeks), and the non-recovery group (patients who were censored before achieving recovery). P-values were generated using the mytable function (method 3) in the moonBook package in R. Analyses were conducted descriptively and all P values are nominal. N = the number of patients who underwent DE after LoR and had outcomes available at both the time of LoR and the first visit after DE.

Abbreviations: AP, abdominal pain; CDAI, Crohn’s Disease Activity Index; CD, Crohn’s disease; DE, dose escalation; LoR, loss of response; PMS, partial Mayo score; SD, standard deviation; SF, stool frequency; RB, rectal bleeding; UC, ulcerative colitis.

## **Table S6:** Immunogenicity status by time to response recovery in patients with Crohn’s disease and ulcerative colitis

| **Pharmacokinetics** | **CD** | | | | **UC** | | | |
| --- | --- | --- | --- | --- | --- | --- | --- | --- |
|  | **Early Recovery** | **Late recovery** | **Non-recovery** | **P value** | **Early Recovery** | **Late recovery** | **Non-recovery** | **P value** |
| **Proportion of ADA positive patient’s n/N(%)** | | | | | | | | |
| Pre-DE | 13/30 (43.3) | 7/9 (77.8) | 6/7 (85.7) | 0.045 | 19/42 (45.2) | 4/8 (50.0) | 6/10 (60.0) | 0.699 |
| Post-DE | 14/34 (45.2) | 5/9 (55.6) | 6/7 (85.7) | 0.150 | 18/43 (41.9) | 4/7 (57.1) | 4/8 (50.0) | 0.716 |
| **Antibody titer (ratio)**  **_among ADA positive** | N=12 | N=5 | N=6 |  | N=16 | N=4 | N=4 |  |
| Pre-DE | 189.0  [63.0-945.0] | 567.0  [189.0-567.0] | 567.0  [189.0-567.0] | 0.710 | 378.0  [63.0-1701.0] | 126.0  [42.0-945.0] | 189.0  [189.0-189.0] | 0.506 |
| Post-DE | 189.0  [126.0-945.0] | 1701.0  [567.0-1701.0] | 567.0  [63.0-567.0] | 0.556 | 189.0  [189.0-1701.0] | 378.0  [126.0-1134.0] | 567.0  [378.0-1134.0] | 0.786 |
| Change from Pre- to Post-DE | 0.0  [0.0-126.0] | 1134.0  [42.0-1134.0] | 0.0  [0.0-378.0] | 0.642 | 0.0  [0.0-63.0] | 84.0  [21.0-252.0] | 378.0  [189.0-945.0] | 0.058 |

Pre-DE refers to the time of LoR, which is the week when patients experience a LoR. Post-DE refers to the first visit after DE, representing the time points for assessing CDAI/PMS following DE, including not only scheduled visits but also unscheduled visits and end of study visits. Patients were classified into three groups based on time to response recovery: the early recovery group (patients who achieved response recovery within 8 weeks following DE), the late recovery group (patients who achieved response recovery after 8 weeks), and the non-recovery group (patients who were censored before achieving recovery). All values are presented as median [IQR] for continuous variables and n (%) for dichotomous variables. P-values were generated using the mytable function (method 3) in the moonBook package in R. Analyses were conducted descriptively and all P values are nominal.

Abbreviations: ADA, antidrug antibody; CDAI, Crohn’s Disease Activity Index; CD, Crohn’s disease; DE, dose escalation; IQR, interquartile range; LoR, loss of response; PMS, partial Mayo score; UC, ulcerative colitis.

## **Table S7:** Albumin level by time to response recovery in patients with Crohn’s disease and ulcerative colitis

| **Albumin (g/L)** | **CD** | | | | **UC** | | | |
| --- | --- | --- | --- | --- | --- | --- | --- | --- |
|  | **Early Recovery**  **(N=29)** | **Late recovery**  **(N=7)** | **Non-recovery**  **(N=6)** | **P value** | **Early Recovery**  **(N=43)** | **Late recovery**  **(N=8)** | **Non-recovery**  **(N=8)** | **P value** |
| Pre-DE | 44.7 ± 4.5 | 46.7 ± 3.1 | 44.5 ± 2.8 | 0.452 | 43.8 ± 3.8 | 43.1 ± 4.5 | 42.8 ± 4.7 | 0.754 |
| Post-DE | 44.2 ± 4.9 | 45.0 ± 3.5 | 40.5 ± 5.6 | 0.228 | 44.4 ± 3.0 | 41.5 ± 5.0 | 41.2 ± 3.8 | 0.015 |
| Change from Pre- to Post-DE | -0.4 ± 2.7 | -1.7 ± 2.2 | -4.0 ± 3.9 | 0.023 | 0.6 ± 3.3 | -1.6 ± 2.4 | -1.5 ± 2.6 | 0.063 |

Pre-DE refers to the time of LoR, which is the week when patients experience a LoR. Post-DE refers to the first visit after DE, representing the time points for assessing CDAI/PMS following DE, including not only scheduled visits but also unscheduled visits and end of study visits. Patients were classified into three groups based on time to response recovery: the early recovery group (patients who achieved response recovery within 8 weeks following DE), the late recovery group (patients who achieved response recovery after 8 weeks), and the non-recovery group (patients who were censored before achieving recovery). All values are presented as mean (±SD) for continuous variables. P-values were generated using the mytable function (method 3) in the moonBook package in R. Analyses were conducted descriptively and all P values are nominal. N = the number of patients who underwent DE after LoR and had outcomes available at both the time of LoR and the first visit after DE.

Abbreviations**:** CDAI, Crohn’s Disease Activity Index; CD, Crohn’s disease; DE, dose escalation; LoR, loss of response; PMS, partial Mayo score; SD, standard deviation; UC, ulcerative colitis.
